# Designing a Novel Clinician Decision Support Tool for the Management of Acute Diarrhea in Bangladesh: Formative Qualitative Study

**DOI:** 10.2196/33325

**Published:** 2022-03-25

**Authors:** Rochelle K Rosen, Stephanie C Garbern, Monique Gainey, Ryan Lantini, Sabiha Nasrin, Eric J Nelson, Nour Elshabassi, Nur H Alam, Sufia Sultana, Tahmida Hasnin, Kexin Qu, Christopher H Schmid, Adam C Levine

**Affiliations:** 1 Center for Behavioral and Preventive Medicine The Miriam Hospital Providence, RI United States; 2 Department of Emergency Medicine Warren Alpert Medical School Brown University Providence, RI United States; 3 Department of Emergency Medicine Rhode Island Hospital Providence, RI United States; 4 Nutrition and Clinical Services Division International Centre for Diarrhoeal Disease Research, Bangladesh Dhaka Bangladesh; 5 Emerging Pathogens Institute University of Florida Gainesville, FL United States; 6 Brown University Providence, RI United States; 7 Department of Biostatistics School of Public Health Brown University Providence, RI United States

**Keywords:** clinical decision support tools, diarrhea management, focus group, formative qualitative research, low- and middle-income countries, mobile phone

## Abstract

**Background:**

The availability of mobile clinical decision support (CDS) tools has grown substantially with the increased prevalence of smartphone devices and apps. Although health care providers express interest in integrating mobile health (mHealth) technologies into their clinical settings, concerns have been raised, including perceived disagreements between information provided by mobile CDS tools and standard guidelines. Despite their potential to transform health care delivery, there remains limited literature on the provider’s perspective on the clinical utility of mobile CDS tools for improving patient outcomes, especially in low- and middle-income countries.

**Objective:**

This study aims to describe providers’ perceptions about the utility of a mobile CDS tool accessed via a smartphone app for diarrhea management in Bangladesh. In addition, feedback was collected on the preliminary components of the mobile CDS tool to address clinicians’ concerns and incorporate their preferences.

**Methods:**

From November to December 2020, qualitative data were gathered through 8 web-based focus group discussions with physicians and nurses from 3 Bangladeshi hospitals. Each discussion was conducted in the local language—Bangla—and audio recorded for transcription and translation by the local research team. Transcripts and codes were entered into NVivo (version 12; QSR International), and applied thematic analysis was used to identify themes that explore the clinical utility of an mHealth app for assessing dehydration severity in patients with acute diarrhea. Summaries of concepts and themes were generated from reviews of the aggregated coded data; thematic memos were written and used for the final analysis.

**Results:**

Of the 27 focus group participants, 14 (52%) were nurses and 13 (48%) were physicians; 15 (56%) worked at a diarrhea specialty hospital and 12 (44%) worked in government district or subdistrict hospitals. Participants’ experience in their current position ranged from 2 to 14 years, with an average of 10.3 (SD 9.0) years. Key themes from the qualitative data analysis included current experience with CDS, overall perception of the app’s utility and its potential role in clinical care, barriers to and facilitators of app use, considerations of overtreatment and undertreatment, and guidelines for the app’s clinical recommendations. Participants felt that the tool would initially take time to use, but once learned, it could be useful during epidemic cholera. Some felt that clinical experience remains an important part of treatment that can be supplemented, but not replaced, by a CDS tool. In addition, diagnostic information, including mid-upper arm circumference and blood pressure, might not be available to directly inform programming decisions.

**Conclusions:**

Participants were positive about the mHealth app and its potential to inform diarrhea management. They provided detailed feedback, which developers used to revise the mobile CDS tool. These formative qualitative data provided timely and relevant feedback to improve the utility of a CDS tool for diarrhea treatment in Bangladesh.

## Introduction

### Background

Mobile technology has had a major impact on the rapid access and transfer of information globally. Today, it is estimated that >5 billion people have mobile devices, with over half of these being smartphones [1]. In the health care sector, smartphones are increasingly used to improve communication between physicians and patients as well as to improve clinical decision-making. With >300,000 mobile health (mHealth) apps available in major app stores, the availability of mobile clinical decision support (CDS) to health care professionals has grown substantially with the increased prevalence of smartphone devices and apps [2,3]. Defined as information systems designed to improve clinical decision-making, traditional forms of CDS range from integration of electronic health records to software apps providing guidelines on a clinical topic [4]. A survey conducted in the United States by the Health Information and Management Systems Society [5] revealed that nearly 90% of providers use mobile devices to engage with patients, whereas a US-based survey analyzing physician information sources [6] found that 72% of physicians use a smartphone or tablet to access drug information and 63% to access medical research.

Although existing CDS systems enable health care professionals to leverage the benefits of technology and information for their clinical practice, many individual, institutional, and technological barriers affect the engagement of clinicians with these new technologies. For instance, in a study conducted in the United Kingdom, physicians found it difficult to integrate mobile CDS into their pattern of work, prompting them to seek alternative sources of CDS [7]. Several studies examining mobile CDS use by physicians in the United Kingdom and United States found that the uptake of mobile CDS was hindered because of their perception that using or adopting such technology included having to choose between suggestions given by mobile CDS and traditionally *trusted* sources, disagreement between information provided by mobile CDS and standard guidelines, and the belief that the use of mobile CDS would be perceived as being unprofessional by the patient [7,8].

Such concerns are not limited to high-income countries (HICs). A cross-sectional study aimed at assessing smartphone medical app use among physicians in Ethiopia found that the perceived usefulness of the app was one of the most notable factors associated with medical app use by physicians along with attitude, internet access, technical skills, and information technology support staff [9]. Furthermore, providers in low- and middle-income countries (LMICs) have expressed willingness to use mHealth tools and perceive such technology as playing an important role in reducing health care barriers [9-11]. A comparative study analyzing the limitations of mobile CDS app adoption and use by clinicians in LMICs versus HICs found that users from LMICs, primarily those who practice on their own in rural settings, used the app more frequently and rated the app as more important for their practice [11]. However, another study has shown that the use of CDS in resource-limited settings was associated with stronger adherence to standard guidelines. More specifically, a randomized controlled trial in Bangladesh found that electronic decision support improved treatment changes that were more consistent with the World Health Organization (WHO) guidelines [12]. However, even with such successes, engagement and implementation of such tools are hindered by limited awareness of mHealth, illiteracy, variable quality of care, and poor network connectivity [10]. In both HICs and LMICs, although providers express interest in integrating mHealth technologies in a clinical setting and have reported positive perceptions toward using mobile CDS as part of everyday practice, many are unconvinced of its overall clinical usefulness for patient outcomes because of the lack of literature on this topic [7,13-15].

### Objectives

Despite the potential to transform health care delivery, much still remains unknown about the clinical usability of mobile CDS from a provider perspective, especially in LMICs. As such, the aim of this study is to describe providers’ perceptions about the utility of a mobile CDS tool that integrates predictive models for dehydration assessment in patients with acute diarrhea in Bangladesh and to seek their feedback on the preliminary components of this CDS tool. Qualitative data were gathered through focus groups, with physicians and nurses working in diverse clinical settings, including specialty research and general public hospitals. In consulting with clinicians, we hope to better adapt, and increase user confidence in, the predictive models and treatment recommendations provided by this tool. By seeking feedback on the tool’s layout and design to ensure that it fits appropriately into different clinical contexts, this formative qualitative research better enables the designers to build a CDS tool that anticipates and addresses the aforementioned barriers.

## Methods

### Study Design and Setting

Qualitative data were collected in a series of focus group discussions (FGDs) from November to December 2020 among clinicians working at 3 distinct hospitals in Bangladesh as part of the *Novel, Innovative Research for Understanding Dehydration in Adults and Kids* (NIRUDAK; which also means *dehydration* in Bangla) study. NIRUDAK is an ongoing research effort to develop diagnostic models and incorporate them in a mobile app to support clinical decisions in the treatment and assessment of dehydration severity in patients with acute diarrhea. The focus groups obtained feedback from nurses and physicians on the clinical utility of the NIRUDAK mHealth app (NIRUDAK app) to understand the current use of mHealth and other CDS tools, understand factors clinicians consider essential in treating patients with diarrhea, review the preliminary app design and content, and seek feedback on app development before a pilot test and trial of its clinical use.

Owing to travel restrictions imposed by the COVID-19 pandemic, all data were gathered using a web-based platform (Zoom; Zoom Video Communications, Inc). Data were collected from clinicians working at three hospitals in Bangladesh: (1) the International Centre for Diarrhoeal Disease Research, Bangladesh’s (icddr,b) Dhaka Hospital; (2) Narayanganj General Victoria District Hospital; and (3) Shaheed Ahsan Ullah Master General Hospital (also known as Tongi Upazilla or Subdistrict Hospital). icddr,b’s Dhaka Hospital is a 350-bed, not-for-profit international research hospital specializing in the treatment of diarrheal illnesses and providing clinical services at no charge to over 100,000 patients with acute diarrhea a year from a catchment area of over 17 million people from the city of Dhaka and its nearby rural districts [16]. Narayanganj General Victoria District Hospital is a 100-bed facility in the town of Narayanganj [17]. Treating 30 to 40 patients with diarrhea per day, this district hospital works as a referral center to primary-level facilities, such as Tongi Upazilla, and is also a site of the nationwide diarrhea surveillance program run by the Institute of Epidemiology, Disease Control and Research and icddr,b. The Tongi Upazilla Hospital, a 250-bed hospital, acts as a primary-level health facility in the district of Gazipur [17].

### Dehydration Management and the NIRUDAK App

Appropriate rehydration with oral and intravenous fluids is the most important treatment for acute diarrhea and requires an accurate assessment of dehydration level [18-24]. Patients with mild to moderate dehydration can be treated with oral rehydration solution in the outpatient setting, whereas those without any dehydration often times need only instructions for management at home [23-25]. Patients with severe dehydration require intravenous fluids in a hospital setting to avoid hemodynamic instability, organ ischemia, and death [23-25]. As the severity of illness can vary greatly among patients, accurate assessment of dehydration status remains a critical step in diarrhea management and can reduce morbidity and mortality that results from both overhydration and underhydration of patients [23-25].

**Figure 1:**
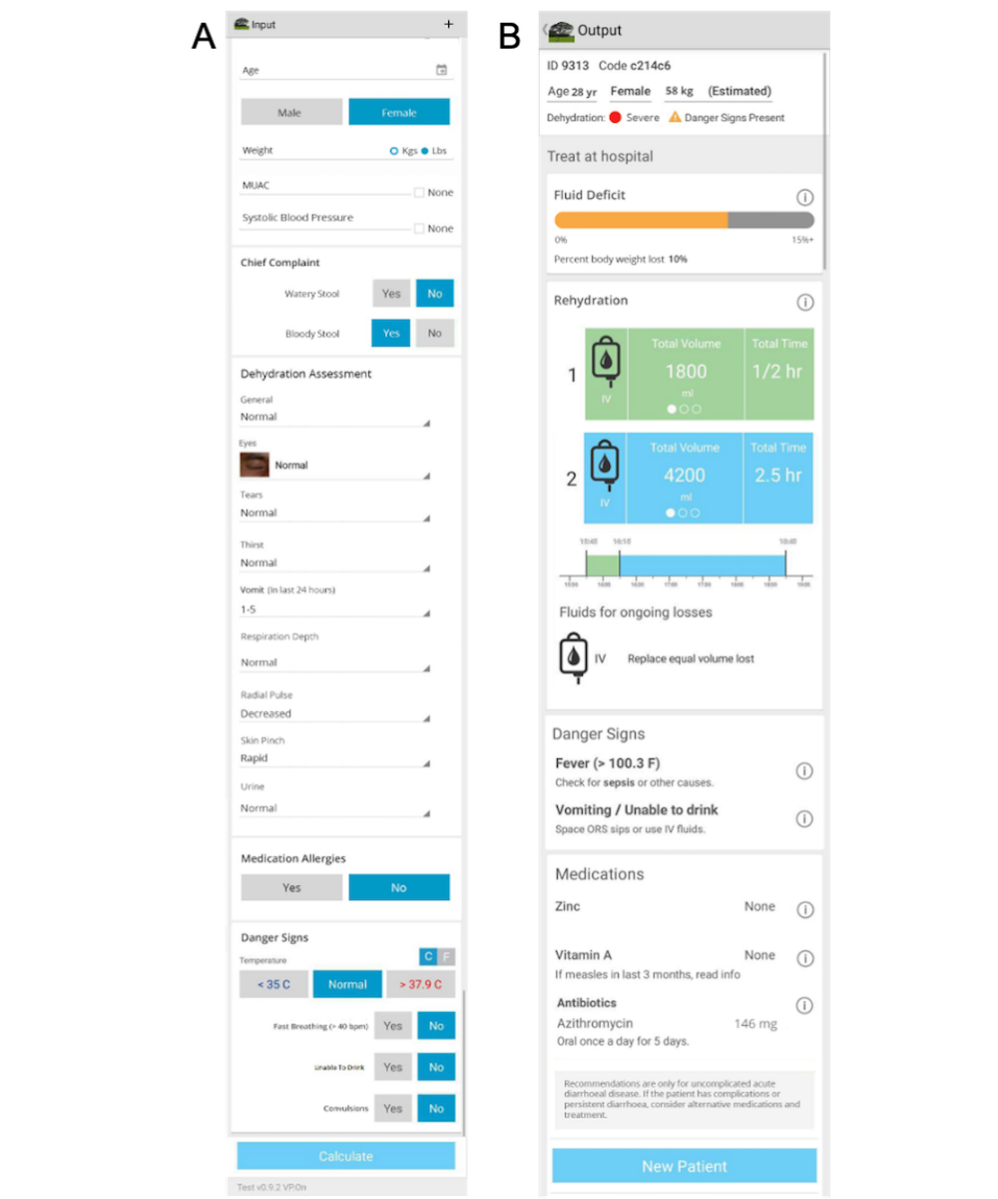
Screenshots of the NIRUDAK app’s input and output screens shown to the participants during focus group discussions. (A) The input screen illustrates where clinicians enter relevant data based on a clinical assessment. (B) The output screen displays the patient’s dehydration severity level and fluid deficit as well as targeted recommendations for rehydration. NIRUDAK: Novel, Innovative Research for Understanding Dehydration in Adults and Kids.

The NIRUDAK app was developed to incorporate several clinical diagnostic models, derived using logistical regression, for assessment of dehydration severity in patients with acute diarrhea aged >5 years (full and simplified NIRUDAK models) and in children aged <5 years (DHAKA [Dehydration: Assessing Kids Accurately] score) [23,26]. On the basis of a review of literature and consultation with expert clinicians at icddr,b, a total of 18 signs or symptoms of dehydration were selected to derive the full NIRUDAK model. A total of 11 more basic clinical predictors were selected for the simplified NIRUDAK model with the intention that it could be used in settings where resources may be limited (ie, places without the ability to measure blood pressure, which is required for the full model) [23]. After assessing each model’s performance, the final full NIRUDAK model included 8 predictors of dehydration, and the final simplified NIRUDAK model included 7 (Multimedia Appendix 1) [23]. The algorithms of both models were then incorporated into a mobile app prototype. The prototype was derived from an mHealth CDS (*Rehydration Calculator*) that adapted paper-based WHO guidelines to the digital medium [27,28]. The prototype allowed for clinicians to enter a patient’s symptoms in the input screen (Figure 1A) and to receive the patient’s dehydration severity level and specific treatment recommendations on the output screen (Figure 1B). Once validated, the NIRUDAK app will enable dehydration severity level assessment (none, some, or severe) and improve the management of patients with acute diarrhea in low-resource settings.

### Study Participants

A total of 8 focus groups were conducted. Of the 8 focus groups, 2 (25%) were conducted with clinicians from each of the district and subdistrict hospitals, one with nurses and the other with physicians. Moreover, of the 8 focus groups, 4 (50%) were conducted with icddr,b providers, 2 (25%) with physicians and 2 (25%) with nurses. Each focus group ranged from 2 to 4 participants, with most focus groups being conducted with 4 participants. The number of participants per focus group was deliberately kept low, in keeping with best practices for remote focus groups [29,30].

### Data Collection

All FGDs were conducted using a web-based platform (Zoom) in Bangla and facilitated by a member of the Bangladesh-based research team. Facilitators used a written focus group agenda, which ensured that all groups were facilitated similarly, and all participants were asked the same series of questions. The agenda asked about the current use of mHealth tools and then presented a standardized case of a patient with diarrhea. Clinicians were asked to identify the patient data essential to determining diarrheal treatment, including rehydration. Facilitators then showed a short video of a prototype app, demonstrating its key features, input screens, and components. Still images of each screenshot were then shown; participants were asked for feedback on input and output screens and to choose between different possible layouts and models for the final mHealth app. Most of the focus groups were approximately 1-hour long, and typed transcripts ranged from 26 to 29 pages. Each discussion was audio recorded for transcription and translation by the local research team, which included the facilitators.

Transcription and translation were conducted in multiple steps and took approximately 8 weeks to complete. First, audio recordings were transcribed into Bangla. Next, another team member reviewed the audio and Bangla transcript to ensure accuracy and that all data had been deidentified. The Bangla transcripts were then translated into English by a research team member proficient in written and spoken English. The English transcripts were reviewed in Bangladesh by a third team member for accuracy. Finally, a US-based research team member read each English transcript to determine if any further clarification was needed. The Bangladesh-based research team reviewed and resolved all translation clarification requests. Once these were addressed, the English transcripts were considered finalized and used in the coding and data analysis process.

### Data Analysis

The research team used applied thematic analysis, a rigorous yet inductive approach designed to identify and examine themes from textual data [31]. Several steps were conducted to augment the rigor and credibility of the qualitative analysis, with coding occurring in 2 major stages.

In stage 1, coding structures were derived inductively as themes and repetitions emerged from reading the first 3 transcripts line by line and systematically categorizing emergent codes. A codebook was created to index and define each emergent code. An audit trial was used to document the iterative process of consolidating and creating emergent codes. In stage 2, the 8 transcripts were independently coded by 2 analysts who then met to compare codes and resolve discrepancies to establish intercoder agreement. Transcripts and codes were entered into NVivo (version 12; QSR International) for analysis [32].

Next, summaries of concepts and themes were generated from reviews of the key aggregated coded data. Coding summaries report participant comments for relevant codes, tracking the number of comments and the distribution of the data among participants. Data in the code summaries were organized by clinician category (nurse or physician) and hospital type (specialty, district, or subdistrict) for easy comparison of similarities and differences among the participants in those categories. Thematic memos, which gathered data from several summaries into key topic areas, were then written and used for the final analysis.

Five qualitative team members participated in the analysis: three coders (the study project coordinator [MG]; a master’s level analyst [RL]; and one of the coinvestigators, a PhD anthropologist [RKR]), who were supported by two coinvestigators with experience in treating diarrhea in Bangladesh (SCG and SN). All data and memos were interpreted in collaboration with both US- and Bangladesh-based research team members and with the principal investigator (ACL) and app designer (EJN; both doctors of medicine with extensive global health and diarrheal disease expertise) for the purpose of identifying themes that explore the clinical utility of an mHealth app to assess dehydration severity in patients aged >5 years with acute diarrhea.

### Ethical Approval and Consent to Participate

Ethical approval for the formative phase of the NIRUDAK study was obtained from the icddr,b (PR-20048) and the Lifespan-Rhode Island Hospital (1624612) institutional review boards.

## Results

### Overview

In total, 8 focus groups were attended by 27 participants. Of these 27 participants, 14 (52%) were nurses and 13 (48%) were physicians; in addition, of the 27 participants, 15 (56%) worked at icddr,b Dhaka Hospital and 12 (44%) worked in government district and subdistrict hospitals. The participants’ experience in their current position ranged from 2 to 14 years, with an average of 10.3 (SD 9.0) years. Additional demographic information is shown in Table 1.

Several key themes emerged from the qualitative data analysis: current experience with CDS, overall perception of the app’s utility and its potential role in clinical care, feedback on specific app details, barriers to and facilitators of app use, considerations of overtreatment and undertreatment, and guidelines for the app’s clinical recommendations. We present these themes in our results and consider the implications of each for the CDS tool development in the discussion that follows.

**Table 1:** Baseline demographics (N=27).

Characteristics				Values
**Age (years), n (%)**				
	25-34			6 (22)
	35-44			16 (59)
	45-54			4 (14)
	55-64			1 (3)
**Sex, n (%)**				
	Men			9 (33)
	Women			18 (66)
**Position and degree, n (%)**				
	**Nurse**			14 (51)
		Diploma	5 (35)	
		Bachelor’s degree	4 (28)	
		Master’s degree	5 (35)	
	**Physician**			13 (48)
		MBBS^a^	8 (61)	
		Master’s degree	5 (38)	
**Monthly household income^b^, n (%)**				
	10,001-50,000 BDT (US $116-580)			7 (25)
	50,001-100,000 BDT (US $581-1160)			6 (22)
	>100,000 BDT (>US $1160)			14 (51)
Experience in current position (years), mean (SD)				10.3 (9.0)
**Hospital location, n (%)**				
	icddr,b^c^			15 (55)
	Tongi Upazilla Subdistrict Hospital			7 (25)
	Narayanganj General Victoria District Hospital			5 (18)

^a^MBBS: Bachelor of Medicine, Bachelor of Surgery (a degree for physicians in Bangladesh).

^b^At the time of the focus group discussion, the US dollar to Bangladesh taka exchange rate was US $1=83.3 BDT.

^c^icddr,b: International Centre for Diarrhoeal Disease Research, Bangladesh.

### Current Experience With CDS

Many, if not all, icddr,b participants (both physicians and nurses) indicated that they had previous experience with CDS and web-based tools. icddr,b clinicians had used SHEBA, an integrated, computerized, and paperless hospital information management system that has been in use since 2009 [33]. The clinical system is installed on all desktops and is the hospital system for patient records. Participants indicated that instructions related to patient follow-up and discharge can be entered into the system as well. District and subdistrict hospital clinicians had some CDS tool experience, including a diarrhea management tool; however, overall, they reported limited practice experience. Many participants, regardless of the setting, mentioned using the Bangladesh Drug Information Management System, a software-based information app used on a mobile phone. Some participants mentioned using UpToDate, an evidence-based clinical resource that includes several medical calculators. Finally, a few participants mentioned using apps for researching literature or for calculations, such as BMI.

### Overall Perception of the App’s Utility and Role in Clinical Care

Most participants were enthusiastic about the NIRUDAK app. Participants felt that it would take some time to learn to use the app but that once learned, the app would be easy to use and have the potential to ease or decrease their workload over time. A participant noted that when they started practicing medicine, patient assessment was tracked on paper and is now being tracked by computer. They noted the following about using this app:

[Using the app] is a matter of time, also learning. We want to assess [dehydration] with less things. By this I mean...having less buttons or features so our work will be easier. If that can happen, that will be good for us.

specialty hospital nurse

To start using a new thing, initially some problems will occur, which is normal. But it’s an excellent app. If anyone uses [this app], it definitely will be beneficial, and it’s easy. Not only doctors but also nurses can use it comfortably and, in many cases, our [work] load will decrease. Yes, [the app] is excellent, I think, it can be used.

government hospital physician

Confidence in the app was explicitly discussed in 2 FGDs with nurses. In these FGDs, the nurses reported enthusiasm for the app and felt confidence in use of the app: “You can send this app to any end of the world without hesitation” [specialty hospital nurse].

Several participants noted that the app and its recommendations should not exist in isolation from the clinician and could not replace clinician experience. For instance, participants noted that clinician decision-making for a patient with diarrhea may be more complex than solely determining the level of dehydration. Other comorbidities, such as diabetes and electrolyte imbalances, are important to recognize and may impact the management of diarrhea and dehydration versus the standardized output from the app. Several participants felt that clinical experience could be relevant to decide when the app use was appropriate; for example, clinicians must first make a determination that patients have dehydration versus sepsis in which fluid management strategies may differ substantially: “[If] I give the total [amount of] fluid [recommended by the app] for a patient with severe dehydration and [the patient also has] sepsis, in that case it will be detrimental for the patient*”* [specialty hospital physician].

### Feedback on Specific App Details

#### Overview

During the focus group, participants reviewed screenshots of the NIRUDAK app prototype, including images of the input and output screens. Here, we present comments from our participants about the following three specific app elements: age, danger signs, and the fluid deficit bar used in treatment recommendations. Each element is described in the next sections, with associated participant comments. Figure 1 provides the images of each component.

#### Age

Patient age is one of the first characteristics on the app input screen (Figure 1A). Although some participants recommended amending the age input field to include years and months rather than a calendar drop-down, most of their comments often focused on relevant treatment differences for young children or geriatric patients. These included the utility of mid-upper arm circumference (MUAC) measurement; availability of equipment for measuring blood pressure in children; and use of age-appropriate antibiotics, zinc, and vitamin A.

#### Danger Signs

The input screen also includes several specific danger signs. Temperature, entered in either degrees Celsius or degrees Fahrenheit, is recorded by choosing one of three radial buttons: <35, normal, and >37.9. A total of two *Yes* or *No* radial buttons were used for each of the following: fast breathing (defined as >40 breaths per minute [bpm]), unable to drink, and convulsions (Figure 1A). Participants found all of these data important for clinical judgment and diarrhea assessment. Suggestions for this screen included adding urine color, using a drop-down menu rather than *Yes* or *No* radial buttons for more precise measurement of ability to drink, and including the presence or absence of epigastric pain and comorbidities, such as diabetes or hypertension. A few others suggested that it would be important to have a means to record ongoing urine output, including the time last urine passed. Regarding the fast breathing field, some participants suggested that the respiratory rate cutoff >40 bpm was high. A participant’s comments also suggested that they misunderstood *bpm* to reference a patient’s pulse rate (ie, beats per minute).

The danger sign output screen provides algorithm-based recommendations using the input data. For example, when high fever is present, the app recommends “Check for sepsis or other causes.” If the patient is vomiting and unable to drink, the app recommends “Space ORS sips or use IV fluids” (see the example in Figure 1B). Participants found the danger sign output important and relevant, with a participant commenting that sepsis cannot be properly diagnosed with the limited information used by the app.

#### Dehydration Assessment, Treatment Recommendations, and the Fluid Deficit Bar

The NIRUDAK app assesses dehydration according to what is entered in the dehydration assessment section of the input page (Figure 1A). Predictors included in this assessment are dependent on the selected NIRUDAK models (Multimedia Appendix 1 [23]) but generally included the following variables: eye level (normal or sunken), radial pulse (normal, decreased, or absent), vomiting in the last 24 hours (none, 1-5, 6-10, or >11 times), respiration depth (normal or deep), and skin pinch (rapid, slow, or very slow). A participant suggested adding the number of times stool has been passed to this section of the input page. Other responses to this screen included that it would take a little bit of time to choose from among the drop-down menu choices but that having the choices helped describe *a full, clear scenario* of the patient. In this discussion, another participant stated:

...*[this app] is a good effort and [for] those who do not know [how to assess dehydration], they, by using the app, can do many things. [For] many people, [being in a] life threatening [situation] can be avoided. It is a great effort and a beautiful process; very good, I like the [app].* [government hospital nurse]

Using the information provided in the dehydration assessment section of the input screen, the output screen indicates whether the patient has *some*, *moderate*, or *severe* dehydration and whether danger signs are present. It also makes a recommendation about whether treatment in a hospital is needed, indicates the percent body weight lost, and provides a horizontal bar indicating the percentage of fluid deficit (Figure 1B). Participants discussed this output screen at length, many indicating that it was helpful and would be easy to use and/or understand. A few participants commented that training would be needed to ensure that users would be familiar with the deficit bar and would understand the output and how to use it in treatment. Other recommendations included increasing the font size as well as color coding the information in red, yellow, and green to draw attention to patient risk level and treatment location. A participant also suggested clearly demarcating lines for the percentages on the fluid deficit bar. When asked to choose between an output screen with the fluid deficit bar and one without it, participants who stated a preference all chose the model with the fluid deficit bar. In 1 focus group, nurses discussed how the dehydration percentages for some, moderate, and severe dehydration are related to the WHO treatment guidelines for acute diarrhea [34]. Participants also discussed the role of weight in the calculation and the output screen, noting that if the patient’s weight was based on an estimate rather than an actual measurement, the deficit bar data could be less useful or even incorrect. When asked if the dehydration assessment output would be helpful, a participant said:

Yes, definitely. Yes, obviously [it] will be helpful because I am getting it absolutely readymade. I do not have to think that much…This is excellent, isn’t it? Excellent, nothing else, absolutely first class.

government hospital physician

### Barriers to and Facilitators of App Use

We asked participants what they thought it would be like to use the NIRUDAK app in clinical practice, who should use the app tool, and what would support app use. Participants identified a variety of potential barriers to and facilitators of app use.

Barriers to use included the time it could take to train clinicians to use the app and the requirement for MUAC and systolic blood pressure (SBP) measurements. MUAC and SBP are used in calculating treatment recommendations in the full NIRUDAK model; however, participants noted that some clinical environments do not have MUAC measurement tape and blood pressure cuffs available:

...many times...digital blood pressure for children are not available. In that case, getting these two things [MUAC, SBP] accurately will be a bit difficult if I want to use it for mass population...I think the things [MUAC, BP] are good, but to use in mass population is a bit difficult.

specialty hospital physician

Similarly, it is not always possible to know if the patient has any medication allergies or another required field, particularly if they are nonresponsive.

Factors that ease or facilitate app use included clinicians’ current experience with and use of other web-based tools, including local electronic health record systems, and familiarity with touch screens and other clinical apps:

As we are used to using the SHEBA app, in that case for us, it will not take so much time [to learn how to use app]. But, at the community level, [there may be patient] rush over there or [limited] manpower. In that case, for them, [app use] might face some problems.

specialty hospital physician

Recognizing that the app could be used in a variety of contexts, we asked what it would be like to use the app via telemedicine during a cholera outbreak and if community health workers (CHWs) could use it. There was a difference of opinion about whether the app could be incorporated into telemedicine. Some participants felt telemedicine use was possible, whereas others cautioned that important symptoms, such as sunken eyes, cannot be properly assessed via telemedicine. Many participants saw the app as useful for quick assessment and diagnosis, relatively easy to learn, and not requiring too much time to use, all of which would make it particularly useful during outbreaks. In contrast, some participants noted that time is further limited by the high numbers of patients requiring treatment during a cholera epidemic. In addition in that context, intravenous rehydration is usually started immediately upon arrival. As such, once rehydration has begun, there could be less need and time for assessment via the app, especially if MUAC and SBP measurements were required. The ability to calculate the recommended treatment without MUAC and blood pressure fields was considered a useful option for this context.

Opinion was divided on whether CHWs could use the app as a CDS tool. Although several participants, of which many were nurses, felt that CHWs would be able to use the app, others—often physicians—cautioned that clinical experience was essential and could be a limiting factor to nonphysician use of the app. Those who felt CHWs could use the app noted that it would make treatment decisions easy:

...to input information will be very easy. It may take 1 minute, or 2 minutes, or 3 minutes. Therefore, if health workers are trained, I think that they all can use this app...to make treatment decision.

government hospital physician

This could be a benefit in areas where physicians are less available. Caveats to CHW use included a concern that they would need to be trained to avoid mistreatment and to specifically observe the danger signs that indicate when hospital-based treatment is necessary.

Participants emphasized the relevance and importance of considering the end user’s clinical expertise and judgment in two main ways: first, clinical experience is needed to support the use of the app through the accurate assessment of clinical signs, and second, the importance of avoiding overreliance on the app for clinical decision-making. Participants recognized that clinicians with varying levels of experience may interact differently with the app and that those with less experience may not recognize situations in which the app is less accurate or when there may be a degree of subjectivity (eg, assessment of clinical signs, such as sunken eyes). Similar comments were made by participants about the use of other formal clinical guidelines, which were felt to be primarily used by less experienced clinicians, whereas more experienced clinicians do not rely on guidelines as heavily: “We really do not treat people by [only using] guidelines in front of us, we [also] use our clinical judgement” [government hospital physician]*.*

### Considerations of Overtreatment and Undertreatment: Guidelines for the App’s Clinical Recommendations

Physicians were shown the WHO’s Integrated Management of Adolescent and Adult Illness (IMAI) guidelines for the classification of dehydration [21]. These use the presence of 2 or more clinical signs to classify dehydration as *no*, *some*, or *severe* and recommend appropriate treatment based on the dehydration classification [21]. Some participants, chiefly those from icddr,b, referenced the use of existing guidelines for the treatment of diarrhea, including the WHO guidelines and DHAKA method [22].

**Figure 2:**
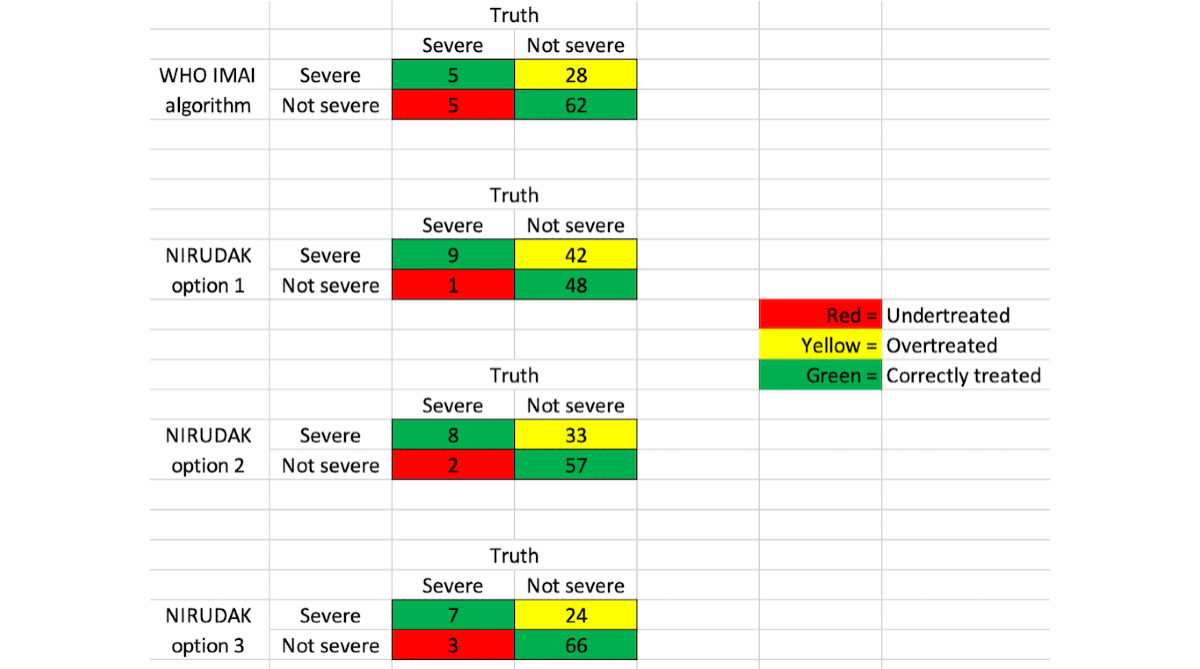
Overtreatment or undertreatment diagram presented to participants during focus group discussions. IMAI: Integrated Management of Adolescent and Adult Illness; NIRUDAK: Novel, Innovative Research for Understanding Dehydration in Adults and Kids; WHO: World Health Organization.

Physicians were also provided with data about the likelihood of correct treatment, overtreatment, and undertreatment of dehydration using the WHO’s IMAI algorithm, and with 3 possible prediction cut points for classifying patients with severe dehydration in the NIRUDAK models (Figure 2). Each of these cut points corresponds to potential sensitivity and specificity thresholds for the NIRUDAK models. Sensitivity refers to the probability that the app will classify a patient as severely dehydrated when the patient is truly severely dehydrated (ie, true positive). Specificity refers to the probability that the app will classify a patient as not severely dehydrated when they are truly not severely dehydrated (ie, true negative) [25,35-37]. As presented in Figure 2, NIRUDAK option 1 illustrates a more sensitive model, selecting cutoffs in which of 100 patients, 57 (57%) would be correctly treated, 1 (1%) would be undertreated, and 42 (42%) would be potentially overtreated. This option avoids undertreating severe dehydration but may misclassify some patients who are not truly severely dehydrated. By contrast, NIRUDAK option 3 illustrates a more specific model, selecting cutoffs in which of 100 patients, 73 (73%) would be treated correctly, 3 (3%) would be undertreated, and 24 (24%) would be overtreated. This third option, compared with the other 2, avoids overtreating nonsevere dehydration but may misclassify some severely dehydrated patients as being nonsevere. Option 2 illustrates a model that falls in between options 1 and 3, neither highly sensitive nor highly specific. Facilitators asked physician participants to discuss their preferences and to weigh the risks and benefits of possibly undertreating severe cases and overtreating cases in which dehydration is not severe.

In general, participants indicated that it is important to avoid both undertreating and overtreating patients. In discussing the importance of not missing cases of severe dehydration, the physicians listed the subjective nature of some of the signs and symptoms used by the app and noted that these differed among very young, middle-aged, and older participants.

Physician preference was generally split between options 1 and 2. There were also no differences between physicians from the two settings regarding option choice; 3 doctors from icddr,b and 4 from government hospitals preferred option 1, whereas 2 physicians from each setting preferred option 2. In addition, 2 participants declined to make a selection or argued that although missing a patient with severe dehydration is problematic, because the patient may die, overtreatment can cause loss and damage as well: “over treatment is as perilous as [being] dehydrated*”* [specialty hospital physician].

## Discussion

### Principal Findings

Participants in the 8 focus groups were mostly enthusiastic about the NIRUDAK app, a novel CDS tool for diarrheal management in low-resource settings. They highlighted the potential for time saving and the utility of the product during high-volume patient periods, such as during a cholera outbreak. Participants’ opinions about key components and the barriers to and facilitators of app use were shared with the coinvestigators and the app design team and informed the next stage of the app development.

Factors that support the use of the NIRUDAK app include clinicians’ familiarity with, and current use of, other mHealth tools, including a touch screen or haptic electronic medical record and other phone-based clinical apps. Notably, discussions about the anticipated utility of the app often occurred during FGDs with nurses, which could reflect a greater role that CDS tools may have for nurses than for physicians. This is an important consideration for the scale-up of the use of the app, given that a large number of patients with acute diarrhea and other common illnesses in LMICs are attended to by nurses or nonphysician health workers working in health centers rather than by physicians in hospital settings [38]. These findings suggest that targeting the use of the app toward nurses or nonphysician clinicians, especially those working in health facilities with lower resources, may allow the app to have the greatest impact.

However, several physicians, cautioned against the use of the app by those with no or little formal clinical training, such as CHWs, versus those with formal clinical training, such as nurses and physicians. Such concerns were due to the possible misuse of the app or that CHWs may not be able to adapt the app’s recommendations to unique cases. Further guidance and training in the use of the app and assessment of clinical signs may be important for the implementation of the app among CHWs and is in line with other recommendations that improving knowledge and skills is essential to improving the quality of care provided in LMICs [39].

**Figure 3:**
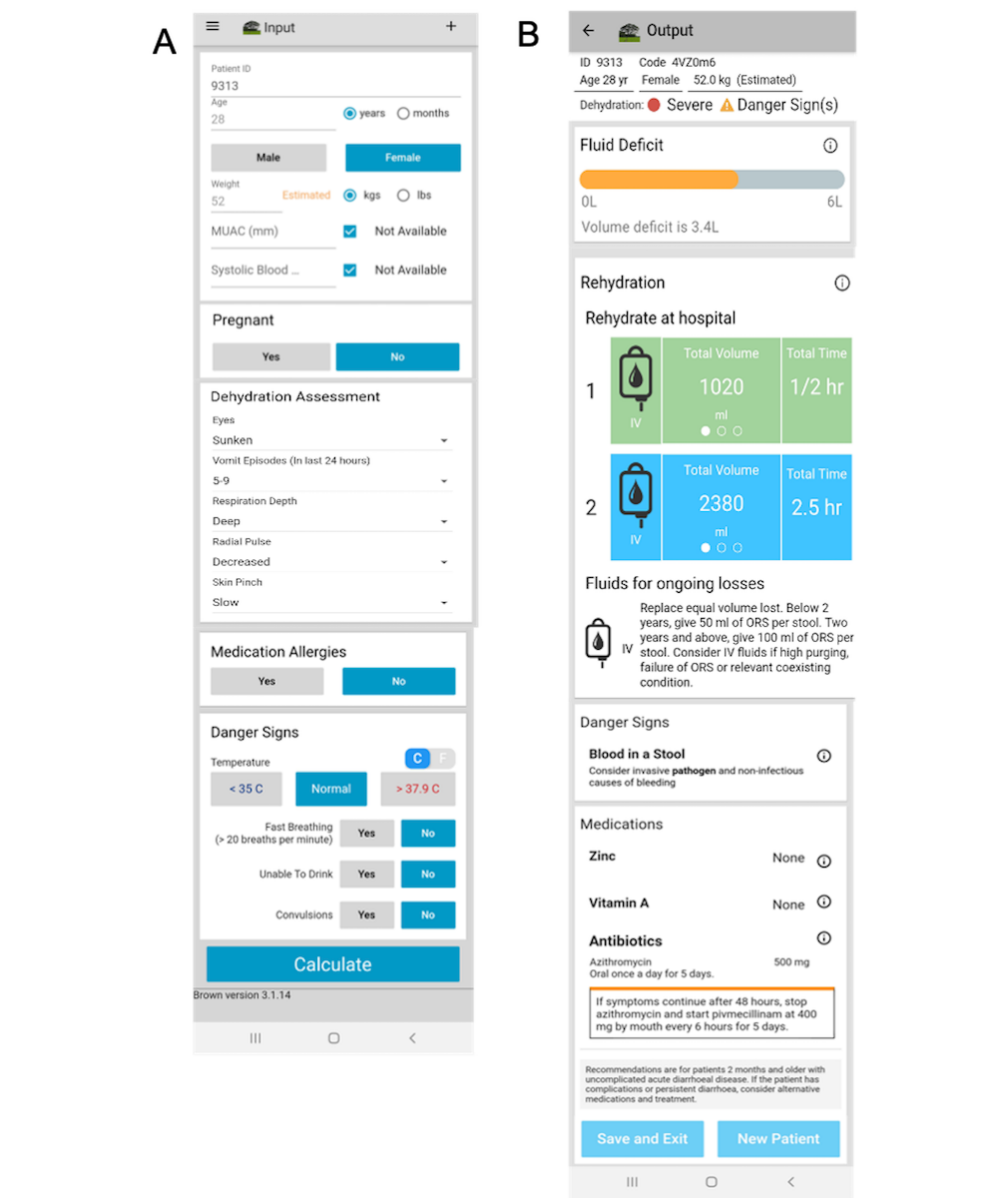
Screenshots of the NIRUDAK app’s (A) input screen and (B) output screen after participants provided detailed feedback on the app. NIRUDAK: Novel, Innovative Research for Understanding Dehydration in Adults and Kids.

The NIRUDAK app uses patient data to provide rehydration and other treatment recommendations based on whether the entered information indicates that the patient has no, some, or severe dehydration. The focus group questions about participants’ use of existing treatment models, specifically the WHO’s IMAI and the DHAKA method, were designed to help the researchers understand clinicians’ opinions and treatment practices and to guide the models used in the NIRUDAK app itself. Of note, the overtreatment and undertreatment models shown in Figure 2 were provided as an aid during the FGDs so that the physicians could visually see how the various prediction cut points potentially used by the app would affect patient treatment. Our participants were concerned about the use of MUAC and SBP as metrics in calculating the rehydration recommendation. Although participants from the diarrheal specialty hospital indicated that these tools are sometimes used and may be available, participants from both the specialty hospital and the government hospitals cautioned that MUAC and SBP measurement will likely be unavailable in government hospitals or in more remote treatment clinics. Although the NIRUDAK app includes separate models based on resource availability, in response to this concern, the developers ensured the app automatically transitions to the simplified model for dehydration assessment when the user selects *Not Available* for either MUAC or SBP (Figure 3A). Such a feature was highly desirable by clinicians and will be appropriate for the app’s use in contexts similar to Bangladesh at both specialty diarrhea and government hospitals. In general, CDS and mHealth tools in LMIC contexts must always consider resource limitations and allow for adaptation within the tool itself depending on resource availability [40].

The app developers made several other changes based on focus group participants’ feedback (Figure 3). The age input field has been amended to include years and months for children aged <5 years. In addition, as 1 participant misunderstood *bpm* on the input page, this acronym was changed to its expanded form *breaths per minute* to prevent further confusion (Figure 3A). The development team was also concerned that the danger signs screen potentially required too much time and considered eliminating it. However, as the participants found it important and relevant, the danger signs section remained on the output screen (Figure 3B). After favorable feedback from participants, the fluid deficit bar, which was also under consideration for elimination, was not only retained but also redesigned in line with participants’ reflections. The participants felt that it provided a necessary visual interpretation to help understand both the severity of a patient’s dehydration and how much fluid they needed as part of their management (Figure 3B). In addition, it is also an improvement over the current WHO guidelines, which do not provide patient-specific guidance on how much fluid to give [21]. Finally, based on the feedback from the participants, option 2 was chosen as the default for predicting dehydration severity. However, because of the concerns expressed by the participants on overtreating or undertreating patients, an additional option was added to the settings menu of the NIRUDAK app that allowed clinicians to switch to the more sensitive option 1, which minimizes undertreatment, or the more specific option 3, which minimizes overtreatment, based on their practice settings and individual patient factors. The fact that there were no patterns of difference or preference between the 2 clinical settings further supported the decision to have both options available in the app.

Participant comments about the varying contexts of use and the varying needs of users indicate that this decision support tool for diarrhea treatment will be used differently depending on the clinician’s role and the clinical context. For nurses and CHWs, the treatment recommendations provided through the app may be directive or proscriptive. For physicians and other advanced practitioners with significant diarrheal treatment experience, the app will support their clinical experience and judgment. Allowing clinicians to adjust the app settings, choosing between a more sensitive, more specific setting and the default setting both preserves the physician autonomy and allows for flexibility, making the app more generalizable to different clinical contexts, including when cholera is epidemic. In addition to the design choice allowing users to flexibly adapt the sensitivity and specificity of treatment recommendations, information tabs, which provide details on the model used, have been added to the app.

### Limitations

These qualitative data about the clinical utility of the NIRUDAK app come from focus groups in which participants were shown still images of the app prototype. Consequently, feedback is limited to opinions about the appearance and content of the app; it is not based on actual use. Although conducting FGDs virtually using the Zoom platform allowed for the completion of data collection during the COVID-19 pandemic, poor internet connectivity prevented 19% (5/27) of the participants (4/5, 80%, were from government hospitals) from attending the focus groups, which may have influenced the findings. Meanings may have been lost in the translation process or interpretation of the data during the analytic process and could have introduced biases. However, the involvement of >1 researcher during the transcription, translation, and coding processes minimizes the likelihood of misinterpreting research findings. In addition, this study was conducted in urban or semiurban hospital settings. Future work should focus on evaluating an mHealth app’s clinical utility in rural or outpatient or ambulatory settings, as well as in other countries, and would be especially valuable.

### Conclusions and Future Directions

The NIRUDAK app has been revised based upon formative qualitative data, which have contributed to the app’s development and programming. The current iteration has been programmed with several features that were influenced by focus group participant feedback, including an option for clinicians to change between 2 different dehydration treatment models. The NIRUDAK app will be field-tested at icddr,b in 2022 to validate those models. Additional qualitative data will also be collected via individual interviews with nurses and physicians who field-test the app to further evaluate NIRUDAK’s usability and understand the clinical users’ experiences.

## Appendix

Multimedia Appendix 1 Predictors included in full and simplified Novel, Innovative Research for Understanding Dehydration in Adults and Kids models [23].

## Abbreviations

bpm: breaths per minute
CDS: clinical decision support
CHW: community health worker
DHAKA: Dehydration: Assessing Kids Accurately
FGD: focus group discussion
HIC: high-income country
icddr,b: International Centre for Diarrhoeal Disease Research, Bangladesh
IMAI: Integrated Management of Adolescent and Adult Illness
LMIC: low- and middle-income country
mHealth: mobile health
MUAC: mid-upper arm circumference
NIRUDAK: Novel, Innovative Research for Understanding Dehydration in Adults and Kids
SBP: systolic blood pressure
WHO: World Health Organization
