# Supplementary material for: Designing a Novel Clinician Decision Support Tool for the Management of Acute Diarrhea in Bangladesh: Formative Qualitative Study
Source: JMIR Hum Factors. 2022 Mar 25;9(1):e33325. doi: 10.2196/33325 (PMC8994146; doi:10.2196/33325)
Supplement: Multimedia Appendix 1 [file humanfactors_v9i1e33325_app1.docx]

Appendix 1: Predictors included in Full and Simplified NIRUDAK Models [23].

| **Predictors** | **Models** | |
| --- | --- | --- |
|  | Full NIRUDAK | Simplified NIRUDAK |
| Age (years) | 🗸 | 🗸 |
| Sex | 🗸 | 🗸 |
| Eye Level | 🗸 | 🗸 |
| Radial Pulse | - | 🗸 |
| Respiration Depth | 🗸 | 🗸 |
| Skin Pinch | 🗸 | 🗸 |
| Vomiting Episodes in 24 Hours | 🗸 | 🗸 |
| Systolic BP (while lying flat) | 🗸 | - |
| MUAC | 🗸 | - |
